# Clinical Relevance of Liquid Biopsy in Melanoma and Merkel Cell Carcinoma

**DOI:** 10.3390/cancers12040960

**Published:** 2020-04-13

**Authors:** Magali Boyer, Laure Cayrefourcq, Olivier Dereure, Laurent Meunier, Ondine Becquart, Catherine Alix-Panabières

**Affiliations:** 1 Laboratory of Rare Human Circulating Cells, University Medical Centre of Montpellier, 34093 Montpellier, France; m-boyer@chu-montpellier.fr (M.B.); l-cayrefourcq@chu-montpellier.fr (L.C.); 2 Department of Dermatology and INSERM 1058 Pathogenesis and Control of Chronic Infections, University of Montpellier, 34090 Montpellier, France; o-dereure@chu-montpellier.fr; 3 Department of Dermatology, University of Montpellier, 34090 Montpellier, France; l-meunier@chu-montpellier.fr (L.M.); o-becquart@chu-montpellier.fr (O.B.)

**Keywords:** cancer, skin cancers, liquid biopsy, biomarkers, melanoma, merkel cell carcinoma

## Abstract

Melanoma and Merkel cell carcinoma are two aggressive skin malignancies with high disease-related mortality and increasing incidence rates. Currently, invasive tumor tissue biopsy is the gold standard for their diagnosis, and no reliable easily accessible biomarker is available to monitor patients with melanoma or Merkel cell carcinoma during the disease course. In these last years, liquid biopsy has emerged as a candidate approach to overcome this limit and to identify biomarkers for early cancer diagnosis, prognosis, therapeutic response prediction, and patient follow-up. Liquid biopsy is a blood-based non-invasive procedure that allows the sequential analysis of circulating tumor cells, circulating cell-free and tumor DNA, and extracellular vesicles. These innovative biosources show similar features as the primary tumor from where they originated and represent an alternative to invasive solid tumor biopsy. In this review, the biology and technical challenges linked to the detection and analysis of the different circulating candidate biomarkers for melanoma and Merkel cell carcinoma are discussed as well as their clinical relevance.

## 1. Introduction

This review discusses the most recent data on liquid biopsy in patients with melanoma, the most common skin cancer with high prevalence in US and European populations [1], or Merkel Cell Carcinoma (MCC), a skin tumor with a disease-associated mortality rate even higher than that of melanoma [2]. Due to their high mortality rate, new technologies are needed to improve the patient outcome. Particularly, specific biomarkers are required to facilitate their diagnosis and management. For a long time, cancer study was based on the analysis of specimens from the primary tumor or its metastases and on imaging data. The current limitations of tumor tissue biopsies and clinical imaging for cancer diagnosis and molecular profiling have led to the development of liquid biopsy. Indeed, tumor biopsy is an invasive procedure, and tumor tissue (especially in patients with cutaneous melanoma) is not always available. Therefore, liquid biopsy, a blood-based analysis of tumor-specific biomarkers, has been introduced as a new diagnostic approach that relies on circulating tumor cells (CTCs) and on circulating tumor-derived factors, such as cell-free tumor DNA (ctDNA), microRNAs (miRNA) and exosomes. The ultimate goal of liquid biopsy is to use the information gathered from such cells and factors to predict early cancer progression and to longitudinally monitor the treatment response, for a personalized medicine of patients with cancer. In melanoma, liquid biopsy has been already used to study many biomarkers (e.g., CTCs, ctDNA and exosomes), and its clinical pertinence is currently investigated in various clinical trials. For MCC, studies are in the early days and very few articles have been published. Moreover, no clinical trial is assessing liquid biopsy in MCC. In this review, we describe the circulating biomarkers and discuss the technical challenges and the clinical relevance of liquid biopsy for these two skin malignancies.

## 2. Melanoma

Melanoma represents only 10% of all skin malignancies, but is one of the most aggressive cancers, responsible for approximately 80% of all skin cancer-related deaths [3]. Risk factors are well known and include acute sun exposure during childhood, teenage and early adulthood, genetic background, skin pigmentation features and number of naevi [4]. Its incidence is steadily increasing in most western countries. Melanoma can be surgically cured if detected at early stages, but survival rates are drastically reduced when discovered at advanced stages. The main issue for melanoma management is the huge heterogeneity of molecular changes that can occur during the disease course. Liquid biopsy might represent a valuable tool especially in high-risk patients with advanced stage melanoma (IIc, III and IV) because currently, no melanoma-specific blood-based biomarker test is available. Biomarkers could be used to provide “real-time” snapshots of the cancer before, during and after specific treatments. Different groups have been working on strategies to monitor melanoma course and therapy responses by measuring circulating markers, such as CTCs, ctDNA and miRNA [5]. Here, we will review the most recent advances. 

### 2.1. Circulating Tumor Cells

Originally, liquid biopsy was developed to study CTCs [6]. CTCs are cancer cells that are released by the primary tumor and/or metastases in the circulation. Due to cancer cell short half-life, CTC detection, count and characterization offer real-time data on the cancer status. Moreover, they can bring insights into the heterogeneity of the melanoma cell population. The detection of Circulating Melanoma Cells (CMCs) was described for the first time in 1991. Since then, the many studies on CMCs from patients with melanoma at different stages and using different detection approaches have given conflicting results [7].

#### 2.1.1. Biology

Melanoma cells do not express epithelial cell adhesion molecule (EpCAM), the classical epithelial cell surface marker that is at the basis of most CTC isolation strategies [8]. Therefore, alternative approaches based on the large size of primary melanoma cells have been developed to isolate CMCs by filtration, and several melanoma-specific cell surface epitopes have been tested for CTC enrichment [9]. Indeed, as metastatic melanoma is a highly heterogeneous tumor, CMCs may display different phenotypes and functional states. For example, nestin, CD133 [10], receptor activator of NF-kB (RANK) [11], ABCB5 [12], CD20 [13] and CD271 [14] have been proposed as potential candidates for the identification of melanoma-initiating cells. However, the diversity of markers limits the possibility to compare studies and reduces the significance of the obtained results.

Melanoma is one of the malignancies which present the highest mutation landscape, mainly caused by carcinogenic ultraviolet (UV) light exposure and other mutational process. The most common genomic alterations studied in melanoma are BRAF, NRAS, TP53, CDKN2A, PTEN, NF1, KIT, RAC1 and TERT [15,16]. Despite the lack of clear clinical relevance of most mutations, some exceptions exist, for example, BRAF V600 mutations clearly predict sensitivity to inhibitors of BRAF and MEK [15]. Interestingly, recent studies suggest that the mutational heterogeneity of melanoma cells might influence their volume and the expression of surface markers. For example, activation of the RAS/RAF pathway drives the expression of HMW-MAA, commonly used as a surface marker for CMC enrichment [17,18]. Georges et al. also reported that the RAS/RAF-mutated cohort present a larger proportion of surface marker-positive cells (e.g., CSPG4/MCAM) compared to the non-RAF/RAS mutated cohort and concluded that the positive enrichment method based on surface markers could be biased by the mutational status of the cells which lead to the loss of subsets of CTCs [19]. On the other hand, treatment with BRAF inhibitors decreases the volume of enlarged BRAF-mutated melanoma cells in a glucose-dependent manner [20]. 

CTC analysis showed not only their genomic heterogeneity and phenotypic diversity, but also their ability to form clusters and to escape the immune system [21]. Indeed, CMCs can be detected as circulating clusters [22]. Studies in other cancer types demonstrated that in clusters, CTCs display a higher metastatic potential with longer survival and reduced apoptosis following dissemination despite a faster clearance [23,24]. Concerning the immune system escape, it is well known that programmed death-ligand 1 (PD-L1)-positive cancer cells are not detected and destroyed by immune cells because its expression hinders their recognition as tumor cells [25]. This observation led to the development of immune checkpoint inhibitors, antibodies against PD-L1 and its receptor PD-1, with a remarkable clinical response in different malignancies, particularly in melanoma.

#### 2.1.2. Technological Challenges

In the first studies on CMCs, reverse transcription-polymerase chain reaction (RT-PCR) techniques were used to amplify different melanoma-specific transcripts. Several studies have showed that CTC detection is associated with disease progression in patients with advanced melanoma [26,27,28,29,30,31]. However, the use of CTC data for the management of patients with melanoma has not been incorporated in the clinical practice, probably because different, non-standardized methodologies were used. 

During the last decade, the CellSearch^®^ system (Menarini Silicon Biosystems Inc), a standardized, US Food Drug Administration (FDA)-cleared methodology for CTC detection (for metastatic breast, colon and prostate cancer) has been intensively evaluated as a prognostic tool in patients with different metastatic solid tumors. A CellSearch^®^ Circulating Melanoma Cell Kit is also available. Despite the limited number of studies on this kit, they all reported similar results: detection of two or more CMCs in approximately 25% of patients with metastatic melanoma, and significant association of CMC detection with overall survival (OS) [9,32,33]. 

In parallel, many other new technologies have been developed to overcome the challenge of CMC detection. Different microfluidic chips and biosensors have been tested in patients with melanoma [34,35,36,37,38,39,40]; however, the multiplicity of procedures reduces their potential clinical utility, like previously observed for RT-PCR-based methods.

Another very new and interesting technology might overcome the low sensitivity of the available CTC assays by analyzing larger blood volumes. For instance, the in vivo photoacoustic flow cytometry platform uses a high pulse rate laser and focused ultrasound transducers for transcutaneous label-free detection of CMCs. This method is called “Cytophone platform”, and detected individual CMCs, clots and CMC-clot emboli in 27 of the 28 patients with melanoma under study [22].

#### 2.1.3. Clinical Relevance

CMCs as liquid biopsy are not routinely used in clinical practice because of the lack of robust and consistent results. As discussed before, the variety of technologies diminish the statistical power of the collected data. Furthermore, reproducible results, like those obtained with the CellSearch^®^ CMC kit, are not yet of clinical interest because they evaluated the association between CMCs and OS only in patients with advanced disease and short survival probability and should be expanded to patients with non-metastatic melanoma.

Most of the previously described technologies have been tested in patient cohorts enrolled in clinical trials to validate their potential clinical application. Among the eleven studies currently registered in the *ClinicalTrials.gov* database (Table 1), five are devoted to the technological validation and OS evaluation in patients with metastatic melanoma. This specific population is often preferred for the validation of new technologies because it is more likely to find high CMC numbers in patients with metastatic melanoma. The other six studies listed in the *ClinicalTrials.gov* database are assessing CTCs as biomarkers for monitoring the therapy response. The goal is to identify a biomarker that can predict therapy failure before clinical relapse. For instance, changes in the number of CTCs might reflect the treatment efficacy.

Indeed, in these last years, several innovative systemic therapies for the treatment of metastatic melanoma have emerged and novel first-line therapies have replaced conventional treatments. The FDA has approved several inhibitors that target the proto-oncogene serine/threonine-protein kinase BRAF mutated at V600E (BRAFV600E) and mitogen-activated protein kinase (MEK), as well as immune checkpoint inhibitors against cytotoxic T-lymphocyte associated protein 4 (CLTA-4) and PD-1 for the management of patients with advanced-stage melanoma [41]. These therapies have greatly improved the OS of patients with advanced disease; however, some patients will not benefit from these treatments, and many will become resistant [42]. For example, although most patients responded well in the beginning of treatment to BRAF inhibitors, a rapid development of drug resistance has been observed while anti-CTLA4 or anti-PD1 treatment demonstrated a durable response but only in a small fraction of patients (20–30% in monotherapy). With the widening of the indications for these therapies, clinicians are often faced with decisions concerning the clinical benefits relative to the risk of adverse events and the costs of these treatments.

Interestingly, a new study evaluated whether PD-L1 expression on CTCs can serve as a predictive biomarker of clinical benefit and response to the anti-PD-1 treatment pembrolizumab on melanoma patients [43]. Their results showed better response rates in patients with PD-L1^+^ CTCs at baseline and an enhanced progression-free survival (PFS) at 12 months for PD-L1^+^ CTC patients compared with PD-L1^−^ CTC patients. These data suggest that PD-L1 expression on CTC may predict response to pembrolizumab in advanced melanoma patients. Even if these results need further validation in a larger cohort of patients, they indicate that liquid biopsy might be a useful tool to stratify patients more likely to respond to immunotherapy.

### 2.2. Circulating Tumor DNA

Among the many biomarkers used in melanoma, ctDNA is already considered a valuable tool for monitoring the therapy response [44,45,46]. For instance, it has been shown that the increase of ctDNA level in the plasma of patients with BRAF-mutated melanoma treated with targeted therapies precedes the detection of relapses by imaging and by clinical evaluation [47]. Moreover, the main explanation for targeted therapy failure is the emergence of new mutations in cancer cells that can bypass the initial molecular targets. Therefore, ctDNA might help to determine the new mutation landscape and to define new personalized therapeutic orientations. Finally, ctDNA can also be a source of genetic material for additional molecular investigations [3], including the longitudinal follow-up of the epigenetic profile during the disease course. 

#### 2.2.1. Biology

The term ctDNA defines short DNA fragments (<166 pb) that are released from cancer cells in the circulation. They are a part of the cell-free DNA (cfDNA) present in plasma, usually released from cells in a state of apoptosis or necrosis. In cancer, the cell turn-over increases and leads to higher cfDNA amounts in plasma [48]. Several studies showed that ctDNA is a good biomarker for the follow-up of patients with metastatic cancer [49,50,51,52]. Indeed, due to it short half-life (about 2 h), it is representative of the real-time molecular changes in the tumor [53] and might alert about the emergence of new mutations. CtDNA has been used as a biomarker in patients with breast [54,55], colon [56] and lung [57,58] cancer, and its presence has been linked to the diagnosis [59,60], prognosis [61,62,63] and also follow-up of the disease [50,64,65,66,67,68]. CtDNA level has been associated with the overall response rate and also with PFS [47,69]. It also reflects the tumor mutational burden [70]. Therefore, ctDNA has a clinical value for cancer surveillance [47,71]. 

#### 2.2.2. Technological Challenges

As plasma contains a huge amount of biological material (e.g., cfDNA from healthy cells, exosomes.), technologies must be very sensitive to detect the low concentration of ctDNA relative to all the circulating DNA released from non-tumor cells. Technologies must also be sensitive enough to detect single-nucleotide mutations present at low frequency in ctDNA. In their review, Diefenbach et al. [72] listed the methods used to detect ctDNA in patients with melanoma and classified them in two main groups: PCR-based and sequencing-based methods. In the first group, droplet digital PCR [73,74] is sensitive, but needs the previous knowledge of the genetic rearrangements [75], like allele-specific PCR and the Beads, Emulsion, Amplification, Magnetics (BEAMing) digital PCR methods [76]. To try to improve detection of ctDNA mutation, such as BRAF or KRAS, some methods were developed, such as the Allele-Specific Locked Nucleic Acid Quantitative PCR (ASLNAqPCR) [77]. This method will block the amplification of wild-type sequences and the mutated sequences will then be increased and detection will be easier. Next-generation sequencing (NGS)-based approaches have the same limitations of PCR-based methods concerning the low abundance of ctDNA compared with standard sequencing samples. Among these methodologies, the Illumina, Thermofisher Ion Torrent and Roche sequencing platforms have already been used for ctDNA analysis in patients with melanoma. The cost of whole-genome and whole-exome sequencing is also a limitation and more targeted sequencing approaches could be envisaged [72]. In addition, as the typical NGS panels target common somatic driver mutations of cancer, some mutations linked to possible further resistance to treatment could also be included, such as those found in the *BRAF* and *NRAS* genes. Their detection could help to adapt the treatment for personalized medicine. To deal with the low abundance of ctDNA in the whole plasma, many rounds of PCR are needed to analyze ctDNA and differentiate it from other DNA sources. However, many PCR cycles could induce amplification mistakes. Some bioinformatic tools were developed to allow to distinguish ctDNA original mutations from PCR mistakes. For example, Duplex Sequencing (DS) based on barcode, integrated digital error suppression (iDES) which combine DS and a second background polishing, based on a healthy donor background model, or also PCR Error Correction (PEC), who discard redundancies on reads after alignment and allow to detect original reads [78]. All these computational tools will help to deal with the technological challenges of low-abundance ctDNA and permit to detect single nucleotide mutations to better adapt medicine for each patient.

#### 2.2.3. Clinical Relevance

CtDNA can help to determine the tumor genetic heterogeneity and can be used as a biomarker for patient follow-up and the early detection of relapse. As ctDNA comes directly from the tumor and can reflect the mutational burden, it could specifically identify therapeutic targets, particularly when the solid tumor is not accessible. Relapse in patients with advanced melanoma (IIc, III and IV) could be monitored by following the ctDNA level. For example, an initial low level of ctDNA harboring the BRAF^V600E^ mutation has been linked to better OS in patients with melanoma, while high level at diagnosis has been associated with shorter PFS and OS. Likewise, low ctDNA level at diagnosis is a good predictor of the response to immunotherapy in patients with advanced disease [76]. Conversely, ctDNA increase during treatment might reflect primary or secondary resistance to that targeted therapy. Moreover, clinical response of metastatic patients treated with PD-1 inhibitors can be monitored by levels of ctDNA, as the level of ctDNA at the initiation can be predictive of treatment response. It has been demonstrated that undetectable ctDNA level at baseline, as well as a decrease > 50% 3 weeks after treatment initiation are associated with better OS and PFS [79,80]. Concerning ctDNA molecular features, mutations in the *BRAF*, *NRAS*, *KIT* and *TERT* genes are considered melanoma-driving mutations and their detection could help to adapt the strategy for patient monitoring. Indeed, tumor progression mostly correlates with an increase of ctDNA with the same mutation, usually BRAF^V600E^ [3].

Currently, 16 clinical trials can be retrieved from the *ClinicalTrials.gov* database using the key words “melanoma” and “circulating DNA”, of which 11 are still open. Among these ongoing studies, six are assessing ctDNA prognostic value (for example, *BRAF*- or *NRAF*-mutated ctDNA), three are evaluating methods for ctDNA quantification and mutation detection and two are monitoring ctDNA level changes over time and their relationship with treatments (Table 2). 

In conclusion, ctDNA is a potential biomarker for the management and follow-up of patients with melanoma, although the optimization and standardization of the detection and analysis methods must be refined to obtain clinically significant results. Indeed, the many different approaches to detect ctDNA introduce experimental bias that prevents obtaining meaningful and robust data on ctDNA clinical relevance. Therefore, the standardization of the methods to detect ctDNA in patients with melanoma is mandatory before its implementation for the routine follow-up of patients as part of personalized medicine. 

### 2.3. Other Circulating Biomarkers

#### 2.3.1. Proteins

Several serum proteins might have diagnostic and prognostic value for melanoma, including lactate dehydrogenase (LDH), S100B and melanoma-inhibiting activity (MIA). However, according to the staging system by the American Joint Committee on Cancer, LDH is the only circulating protein with significant prognostic value in melanoma [81]. Specifically, elevated LDH concentration in patients with stage IV melanoma correlates with poor survival [82], and is a clinically significant factor associated with response, PFS and OS in patients treated with targeted [83] and immune therapies [84,85]. However, it is of clinical interest only for patients with metastatic melanoma.

Among the proteins expressed and released by melanoma cells, the S100 family is the most studied [86,87]. S100B expression is increased in melanoma cells compared with melanocytes [82], and can be used for the staging of metastatic malignant melanoma by immunohistochemistry [88]. Moreover, serum S100B level is increased in patients with melanoma, independent of the cancer stage [89,90]. Its expression is clearly correlated with the presence of metastases, tumor burden, prognosis and survival [91,92]. S100B could also serve as a strong baseline marker of OS in patients with melanoma receiving anti-CTLA4 and/or anti-PD-1 antibodies [93,94].

MIA is a soluble protein expressed by malignant melanoma cells [95]. This protein was proposed as a diagnostic serum marker of melanoma progression because the MIA ELISA (Enzyme-linked immunosorbent assay) could correctly classify 100% of the investigated serum samples of patients with stage III and stage IV melanoma [96]. However, in a study that compared different serum proteins in 373 patients with melanoma, serum S100B showed the highest sensitivity for newly diagnosed metastases (0.86), followed by MIA (0.80), LDH (0.48) and albumin (0.15). Conversely, MIA displayed the lowest specificity (0.62) compared with albumin (0.99), LDH (0.98) and S100B (0.91) [97]. Similar results were reported by a more recent study in patients with stage II melanoma [98]. Therefore, MIA does not offer more advantages compared with S100B and LDH.

#### 2.3.2. Circulating MicroRNAs

Circulating miRNAs are emerging as potential non-invasive biomarkers for melanoma. miRNAs are directly released in the blood circulation during tumor cell apoptosis or necrosis, but also by cells via extracellular vesicles including exosomes, micro-vesicles and apoptotic bodies, which prevent their degradation by serum and plasma RNases [99]. In the blood, circulating miRNAs are associated with lipid particles, and/or are bound by protective proteins, such as argonaute-2 (AGO2) and nucleophosmin. Therefore, circulating miRNAs are very stable. Although miRNAs are present at extremely low concentrations in the circulation, they can be detected by standard techniques, including real-time quantitative RT-PCR.

During the last decade, it has become increasingly clear that miRNA expression dysregulation in human malignancies directly contributes to the acquisition of cancer hallmarks [100]. Indeed, miRNAs play a critical role in the regulation of many cancer-relevant processes, such as cell proliferation, migration and apoptosis, by regulating the expression of oncogenes (tumor-suppressor miRNAs) and tumor-suppressor genes (oncogenic miRNAs). 

Several reviews have highlighted the role of miRNAs as potential diagnostic and prognostic biomarkers and as key molecular regulators in melanoma development [101,102,103]. For example, miR-137 is a well-established tumor suppressor miRNA often downregulated in melanoma and in many other cancer types. Its downregulation has been associated with poor prognosis in patients with melanoma [104]. This is not surprising because miR-137 inhibits invasion and migration of melanoma cell lines by directly targeting oncogenes, including the transcription factors TBX3, EZH2, c-MET and Y box-binding protein 1 (YB1) [105,106]. 

Several efforts have been made to identify circulating miRNAs that may be used as diagnostic and prognostic biomarkers for melanoma; however, due to the variety of profiling platforms and inputs, and the different techniques for serum and plasma preparation, RNA extraction, quality control, normalization and statistical evaluation, the results of the different studies show limited consistency.

#### 2.3.3. Exosomes

Melanoma cells produce various types of extracellular vesicles (EV), including micro-vesicles, apoptotic bodies and exosomes. The specific EV content and role in recipient cells depend on their molecular composition that is determined by their cell of origin. Cell type-specific proteins, lipids and nucleic acids can be detected in the respective EV populations, and this explains their prognostic and diagnostic value in specific conditions, including different cancer types [107]. Currently, few markers (i.e., TSG101, syntenin and the simultaneous expression of three tetraspanins (CD9, CD63 and CD81)) allows for distinguishing exosomes from other EVs, such as micro-vesicles and apoptotic bodies [108]. It is thought that exosomes secreted by cancer cells have critical roles in several tumor-related biological processes by promoting (1) survival and growth of the primary tumor through cell–cell communications between tumor and non-tumor cells, (2) tumor invasion through extracellular matrix remodeling and (3) angiogenesis [109]. Tumor-derived exosomes may also modulate the immune cell behavior, by dampening the anti-tumor immune response and promoting melanoma progression [109]. Conversely, exosomes secreted by immune cells may modulate melanoma cell behavior and exert therapeutic effects [103,110].

Melanoma-derived and other EVs are generally isolated using established differential ultracentrifugation methods. This enables the separation of different EV types based on their sedimentation rate [111,112]. Other techniques, such as density gradient, precipitation, filtration, size-exclusion chromatography and immunological separation, have been employed with relative success in terms of EV recovery and specificity [113]. Then, electron microscopy, ELISA, flow cytometry and Nanoparticle Tracking Analysis (NTA) approaches are the most commonly used methods for the detection and quantification of exosomes [114,115]. Moreover, microfluidic chips also have the potential to be an emerging tool for exosome separation as well as detection applications with the improvement of using only a single chip for both steps [116]. However, at this stage, there is no recommended isolation/detection protocols and more comparative studies are needed.

Clinically, EVs might become biomarkers of cancer progression, particularly for predicting and, hopefully, preventing future metastasis development, and also therapeutic targets. Hoshino et al. showed that the integrin expression profile of circulating plasma exosomes isolated from patients with cancer directs their tissue- and organ-specific colonization for metastasis [117]. Thus, it will be of interest to target the integrins expressed by these exosomes to prevent metastasis formation. Exosomes could also be used to develop new drug delivery strategies. Indeed, due to their ability to reach a specific tissue, exosomes are promising nano-vehicles for the bio-delivery of therapeutic RNAs, proteins and other agents [109]. Moreover, some recent studies have evaluated the role of exosomal PD-L1 expression in melanoma patients treated with immunotherapy [118,119] and provided a rationale for the application of exosomal PD-L1 as a biomarker to predict therapy response and clinical outcome.

### 2.4. Conclusion

Liquid biopsy in melanoma has already showed its clinical relevance for the early diagnosis, prognosis and follow-up of the disease. However, method standardization needs to be optimized to increase the clinical use and the clinical benefits of the biomarkers assessed by liquid biopsy. Finally, to reduce the costs linked to tumor surveillance and monitoring, data obtained by liquid biopsy might be used in the future to detect relapse before positron-emission tomography/computed tomography (PET/CT) imaging (the current detection approach in patients with advanced melanoma).

## 3. Merkel Cell Carcinoma

MCC is a rare skin cancer that usually appears as a pink/red, rapidly growing skin nodule on UV-exposed areas, such as head, neck and upper limbs. Usually, it is characterized by aggressive behavior and high metastasis rate without specific location [120]. Despite the currently low (but rapidly increasing) incidence (0.7 per 100,000) [121,122,123], MCC is associated with shorter disease-free and OS and higher cancer-related death rates than melanoma. To date, two main different oncogenic pathways have been identified [124,125]. The first is related to UV exposure with high tumor mutational burden, while the second one is related to a ubiquitous DNA virus, Merkel Cell Polyomavirus (MCPyV). Although the involvement of this virus in MCC development has been clearly established, the underlying molecular mechanisms have not been fully characterized. MCPyV was first described in 2008 by Feng et al. [126], and is the first human polyomavirus clearly linked to a human cancer [127]. Moreover, its epidemiological link with immunosuppressive conditions, including chronic lymphocytic leukemia and solid organ transplantation, is well established [128,129].

The hypothesis that MCC originates from epidermal Merkel cells [130] is supported by some common features between Merkel and MCC cells, such as the presence of a cytokeratin network as a dot, and the expression of cytokeratin-20 and neuron-specific enolase [131,132]. However, alternative theories have been proposed, involving, for example, a common cell ancestor with B lymphocytes [133]. 

Besides these debates on MCC cellular origin [131,134], recent studies tried to better understand the mechanisms underlying this malignancy, notably the involvement of the MCPyV virus that is detected in 80% of cases [124,127,135,136,137]. Currently, it is known that MCPyV is first present in cells in an episomal conformation [138] and is subsequently integrated in the cell DNA.

As this tumor remains poorly understood, liquid biopsy might help to decipher its nature, the underlying mechanisms and might ensure a real-time follow-up of the disease and of its response to different treatments. Research on this topic is in its early days, but some studies have already investigated different circulating biomarkers in MCC (Table 3).

### 3.1. CTCs and Circulating miRNAs

The few studies on this topic show how little is known about MCC. Moreover, the existing data concern small patient cohorts, due to MCC rarity. Nevertheless, some circulating biomarkers might help to better understand MCC. CTCs have been associated with patient survival and MCC aggressiveness [139,140,143,145], and high miRNA-375 concentration in plasma with tumor burden [142,146]. Therefore, they are candidate biomarkers for MCC follow-up. In patients with MCC, CTCs are usually detected using the CellSearch^®^ system [147,148,149], based on the positive enrichment of EpCAM-expressing cells. A new CTC detection method based on negative enrichment was investigated by Boyer et al. [144]. They evaluated CTC number to follow the disease course, and also characterized MCC CTCs (e.g., PD-L1 status). They found that CTC detection was associated with the cancer stage. The few studies on circulating miRNA in MCC used RT-qPCR as detection technology [142,150], like for other cancers [151,152]. The MCC miRNome has been investigated mostly in formalin-fixed paraffin-embedded tumor samples [153,154], and the miRNAs identified as MCC-specific could now be evaluated in liquid biopsies. 

### 3.2. Exosomes

Exosomes are small vesicles secreted by different types of cells under the influence of cellular conditions and environment [155]. They might be used as an MCC biomarker. However, they have been investigated only in MCC cell lines and more data are needed. These preliminary studies suggest that exosomes might be a good candidate biomarker. Indeed, they showed that MCC-derived exosomes transport proteins linked to cancer, such as LDH and factors implicated in the p38 MAPK and Wnt signaling pathways. Importantly, these proteins were detected independently of the cell line MCPyV status [155], thus they could be used to monitor all patients with MCC.

### 3.3. Anti-MCPyV Antibodies

The presence of MCPyV in most MCC specimens and the higher incidence in immune-deficient patients indicate the implication of the immune system in MCC [123,124]. The large T (LT) and small T (sT) antigens of MCPyV are involved in oncogenesis, for example their presence has been linked to cell cycle disturbance or viral replication. Moreover, LT is required for the survival of cancer cell lines [156]. Titration of the VP1 capsid protein of MCPyV has been used as a circulating biomarker of the viral load in patients. In these studies, high levels of anti-VP1 and anti-sT antibodies in blood was correlated with better outcome [157], and anti-LT antibodies are a prognostic factor of recurrence if they are detected more than one year after diagnosis [141].

### 3.4. Immunotherapy

An immunohistochemical analysis of MCC specimens found that many cancer cells express PD-L1, particularly when they are in close proximity to infiltrating immune cells [158]. This mechanism is used by MCC cells to escape immunity. Specifically, cancer cells express PD-L1 at their surface, and its interaction with PD-1 at the surface of immune cells will block their identification as cancer cells. This phenomenon has been highlighted in many different cancers and is one of the immune checkpoints targeted by immunotherapy. Recently, immunotherapy based on PD-1/PD-L1 inhibition [159,160,161] has been approved by the US FDA for patients with metastatic MCC. This therapy has already been used in other cancers for some years [162] and is also efficient in patients with metastatic MCC, although the response rate remains unsatisfactory [159,163]. Detection of PD-L1 at the surface of CTCs could help for MCC patient management. This emphasizes the need of better understanding this disease to develop more appropriate treatments. 

A query of the *ClinicalTrials.gov* database with the keyword “Merkel cell carcinoma” in November 2019 did not retrieve any ongoing study on circulating biomarkers in MCC. Most of the listed studies were testing new treatments. 

### 3.5. Conclusion

Liquid biopsy in MCC could be of clinical interest for patient management, as suggested by the correlation of CTCs and circulating miRNAs with disease outcomes and tumor burden. However, more research must be done in larger cohorts and on different potential candidate biomarkers. 

## 4. Discussion

**Figure 1:**
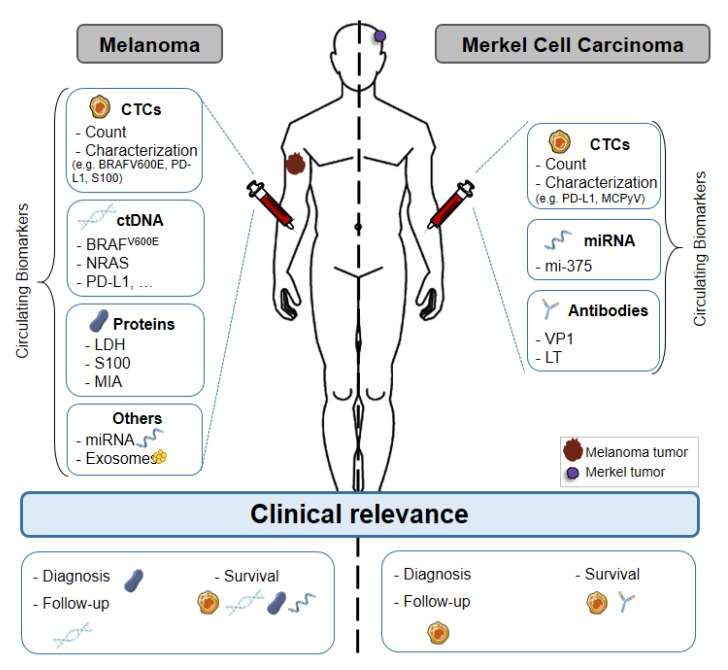
Liquid biopsy of circulating biomarkers in melanoma and Merkel cell carcinoma. Circulating biomarkers used in melanoma and Merkel cell carcinoma: use, characterization and clinical relevance. Abbreviations: CTC: Circulating Tumor Cell, ctDNA: Circulating tumor DNA, miRNA: microRNA, MAPK: Mitogen-activated protein kinases, TERT: telomerase reverse transcriptase, LDH: Lactate dehydrogenase, MIA: Melanoma Inhibitory Activity, VP1: Capsid Protein, LT: Large T antigen, PD-L1: Programmed death-ligand.

**Figure 2:**
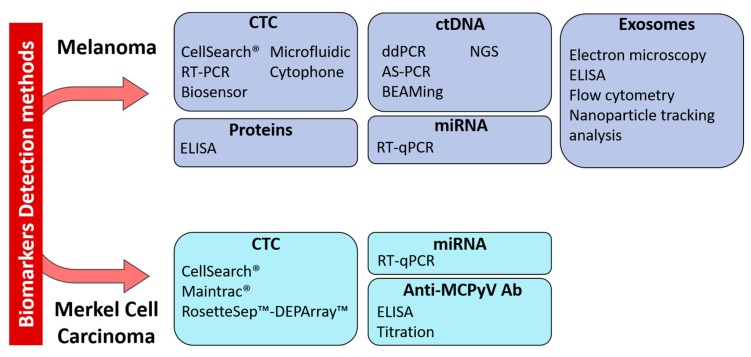
Technologies for the detection of circulating biomarkers currently used in melanoma and Merkel cell carcinoma. Abbreviations: CTC: Circulating Tumor Cell, ctDNA: Circulating tumor DNA, miRNA: microRNA, RT-PCR: reverse-transcriptase polymerase chain reaction, RT-qPCR: reverse-transcriptase quantitative PCR, ELISA: enzyme-linked immunosorbent assay, ddPCR: droplet digital PCR, AS-PCR: Allele Specific PCR, BEAMing: Bead Emulsion Amplification Magnetic, NGS: Next-Generation Sequencing.

The potential use of liquid biopsy in melanoma has already been extensively studied and some circulating biomarkers are clinically relevant (Figure 1). Conversely, few but encouraging data are available in the context of MCC, due to its rarity. The detection of circulating biomarkers in blood is challenging, but technological advances help to deal with their scarcity in liquid biopsies (Figure 2). Circulating biomarkers help to assess the tumor heterogeneity in real-time, unlike conventional biopsy that is representative only of the sampling site. Therefore, liquid biopsy could be clinically relevant in these two skin cancers, for prognosis and staging, and also for the follow-up of patients (Figure 1). In the future, information gained from liquid biopsies might be used to indicate when restaging is needed, or when surveillance is sufficient for patients without evidence of micro-metastatic tumor burden in the blood. Recent studies have shown that the micro-metastatic tumor burden is often increased before the clinical evidence of metastasis by imaging. In the future, both therapy and imaging decision-making might be guided by the data obtained by liquid biopsy. Liquid biopsy could become a really useful tool for the personalized management of patients with melanoma, among whom relapse and resistance to immunotherapy are common, or with MCC, where the risk of aggressive disease is very high. Many studies have proven the value of liquid biopsy in melanoma. On the other hand, clinical studies must be performed in patients with MCC to confirm the relevance of the circulating biomarkers tested in small patient cohorts. Some multi-national projects have been established, such as the European Liquid Biopsy Society (ELBS) or the International Society of Liquid Biopsy (ISLB), to expand the use of circulating biomarkers. 

## Figures and Tables

**Table 1:** Circulating Tumor Cells (CTCs) in melanoma: clinical studies listed in the *ClinicalTrials.gov* database.

N°	Status	Study Title	Cancer	Location	Outcomes	Measurements
1	Completed	Culture and Characterization of Circulating Tumor Cells (CTC) in Melanoma and Other Cancers	Melanoma and other cancers	Comprehensive Cancer Centers of Nevada	Technological validation	CTC culture and analysis
				Las Vegas, Nevada, United States	Survival evaluation	
2	Completed	Study of Circulating Tumor Cells Before and After Treatment in Patients with Metastatic Melanoma	Metastatic Melanoma	CHU of Nice, Nice, France	CTC evaluation from pre- to post-treatment	CTC analysis
					Survival evaluation	Treatment follow-up
3	Recruiting	Biomarker Analysis Using Circulating Tumor Cells in Patients with Melanoma	Melanoma Stage I-IV	Abramson Cancer Center of the University of Pennsylvania	CTC evaluation during treatment	CTC analysis
				Philadelphia, Pennsylvania, United States		Treatment follow-up
4	Completed	Circulating Tumor Cells and Melanoma: Comparing the EPISPOT and CellSearch Techniques	Metastatic Melanoma	CHU of Montpellier, Montpellier, France	Technological validation	CTC analysis
				CHU of Nîmes, Nîmes, France	Survival evaluation	
5	Recruiting	In Vivo Real-time Detection of Circulating Melanoma Cells	Melanoma Stage I-IV	University of Arkansas for Medical Sciences	Technological validation	CTC analysis
				Little Rock, Arkansas, United States		
6	Recruiting	Ex Vivo Expansion of Circulating Tumor Cells as a Model for Cancer Predictive Pharmacology	Melanoma	Saint-Louis Hospital	Therapeutic response	CTC culture and analysis
			Stage III–IV	Paris, France	Survival evaluation	
7	Unknown ^†^	Concurrent Ipilimumab and Stereotactic Ablative Radiation Therapy (SART) for Oligometastatic But Unresectable Melanoma	Melanoma Stage III–IV	Comprehensive Cancer Centers of Nevada Las Vegas, Nevada, United States	Therapy and Survival evaluation Safety and Tolerability evaluation	Gene mutations, serum markers and CTC analysis
						Treatment follow-up
8	Active, not recruiting	Molecular Characterization of Advanced Stage Melanoma by Blood Sampling	Metastatic Melanoma	CHU of Reims	Technological validation	ctDNA and CTC analysis
				Reims, France	Survival evaluation	
9	Recruiting	Lymphodepletion Plus Adoptive Cell Transfer with or Without Dendritic Cell Immunization in Patients With Metastatic Melanoma	Metastatic Melanoma	University of Texas MD Anderson Cancer Center	Therapy and Survival evaluation	T cells and CTC analysis
				Houston, Texas, United States		Treatment follow-up
10	Completed	High-activity Natural Killer Immunotherapy for Small Metastases of Melanoma	Metastatic Melanoma	Fuda Cancer Institute of Fuda Cancer Hospital	Therapy and Survival evaluation	Serum markers, lymphocytes and CTC analysis
				Guangzhou, Guangdong, China		Treatment follow-up
11	Unknown ^†^	Circulating Melanoma Cells in Metastatic Patients Treated with Selective BRAF Inhibitors	Metastatic Melanoma	Istituto Oncologico Veneto IRCCS Padova, Italy	CTC evaluation during treatment	CTC Analysis
					Survival evaluation	Treatment follow-up

CHU: University hospital center, CTC: circulating tumor cell, ctDNA: circulating tumor DNA.

**Table 2:** CtDNA in melanoma: clinical studies listed in the *ClinicalTrials.gov* database.

N°	Status	Study Title	Cancer	Location	Outcomes	Measurements
1	Completed	Circulating Cell-free DNA in Metastatic Melanoma Patient: Mutational Analyses in Consecutive Measurement Before and After Chemotherapy	Metastatic melanoma	CHU of Nice Nice, France	Therapeutic response	ctDNA mutational burden analysis
2	Completed	A Study to Detect V-Raf Murine Sarcoma Viral Oncogene Homolog B1 (BRAF) V600 Mutation on Cell-Free Deoxyribonucleic Acid (cfDNA) from Plasma in Participants with Advanced Melanoma	Metastatic melanoma	UZ Brussel, Brussel, Belgium Institute Jules Bordet, Brussel, Belgium CHIREC Edith Cavell, Brussel, Belgium (and 11 more...)	Therapy response duration	BRAF mutation measurement
					Survival evaluation	

3	Active, not recruiting	Detection of Plasmatic Cell-free BRAF and NRAS Mutations: a New Tool for Monitoring Patients with Metastatic Malignant Melanoma Treated with Targeted Therapies or Immunotherapy (MALT)	Melanoma stage III–IV	CHU of Nice Nice, France	Technological validation ctDNA evaluation during treatment	BRAF and NRAF mutation measurement Measure follow-up
4	Completed	Use of Exome Sequence Analysis and Circulating Tumor in Assessing Tumor Heterogeneity in BRAF Mutant Melanoma	BRAF-mutated Melanoma	Princess Margaret Cancer Centre Toronto, Ontario, Canada	ctDNA evaluation	ctDNA pre- and post-mortem and metastases analysis
5	Recruiting	Biomarkers for the Activity of Immune Checkpoint Inhibitor Therapy in Patients with Advanced Melanoma	Metastatic melanoma	UZ Brussel Jette, Brabant, Belgium		Treatment follow-up
6	Active, not recruiting	Vemurafenib and Cobimetinib Combination in BRAF Mutated Melanoma with Brain Metastasis	Metastatic melanoma	CHU of Bordeaux, Bordeaux, France CHU Ambroise Paré, Boulogne, France CHU Brest Hôpital Morvan, Brest, France (and 14 more...)	Therapy and Survival evaluation	Treatment follow-up ctDNA mutation rate
7	Recruiting	CAcTUS—Circulating Tumor DNA Guided Switch	Metastatic melanoma	The Christie NHS Foundation Trust Manchester, United Kingdom	Therapeutic response	ctDNA level measurement Treatment follow-up
8	Active, not recruiting	Low-Dose Ipilimumab With Pembrolizumab in Treating Patients with Melanoma that has Spread to the Brain	Metastatic melanoma and other cancers	MD Anderson Cancer Center Houston, Texas, United States	Therapy and Survival evaluation	ctDNA level measurement
9	Recruiting	Therapeutic Drug Monitoring of BRAF-Mutated Advanced Melanoma	Metastatic melanoma	Hôpital de Mercy, Ars-Laquenexy, Fr CHRU Nancy, Vandœuvre-lès-Nancy, Fr Institut de Cancérologie de Lorraine (ICL), Vandœuvre-lès-Nancy, Fr	Therapy and ctDNA evaluation	ctDNA level measurement Treatment follow-up
10	Recruiting	Bevacizumab and Atezolizumab with or without Cobimetinib in Treating Patients with Untreated Melanoma Brain Metastases	Metastatic melanoma	MD Anderson Cancer Center Houston, Texas, United States	Therapy evaluation	ctDNA level measurement
11	Active, not recruiting	Molecular Characterization of Advanced Stage Melanoma by Blood Sampling	Metastatic melanoma	Chu of Reims Reims, France	Biomarkers significance	ctDNA analysis
12	Recruiting	Liquid Biopsy Evaluation and Repository Development at Princess Margaret	Cancer or high risk of cancer	Princess Margaret Cancer Centre Toronto, Ontario, Canada	Protocol development	ctDNA analysis
13	Active, not recruiting	Clinical Trial to Evaluate the Efficacy of Vemurafenib in Combination with Cobimetinib (Continuous and Intermittent) in BRAFV600-Mutation-Positive Patients With Unresectable Locally Advanced or Metastatic Melanoma	Melanoma stage III-IV	Hospital Universitario Donostia, San Sebastián, Guipuzcoa, Spain Hospital General Universitario Santa Lucía, Cartagena, Murcia, Spain Hospital Clínic de Barcelona, Barcelona, Spain (and 15 more...)	Therapy and Survival evaluation	ctDNA analysis
14	Active, not recruiting	Selection Pressure and Evolution Induced by Immune Checkpoint Inhibitors and Other Immunologic Therapies	Neuroendocrine metastatic tumors	Princess Margaret Cancer Centre Toronto, Ontario, Canada	Therapy and ctDNA evaluation	ctDNA analysis
15	Recruiting	Circulating Tumor DNA Exposure in Peripheral Blood	Cancer stage 0 - IV	University of Arizona Cancer Center, Tucson, Arizona, US Florida Hospital Celebration Health, Celebration, Florida, US Orlando Health UF Health Cancer Center, Orlando, Florida, US (and 3 more...)	Protocol development	ctDNA level measurement and analysis

CHU: University hospital center, UZ: Universitair Ziekenhuis, NHS: National Health Service, CTC: circulating tumor cell, ctDNA: circulating tumor DNA, Fr: France, US: United-States, UF: University of Florida.

**Table 3:** Studies on liquid biopsy in Merkel cell carcinoma.

Study	Title	Bio-Marker	Inclusion Criteria	*n*	Detection Method	Prognosis Relevance	Ref
Blom et al. (2014)	Clinical utility of a circulating tumor cell assay in Merkel cell carcinoma	CTCs	Stage I–IV	34	CellSearch—Epithelial kit	CTCs associated with survival, prediction of treatment response, shorter OS and reflect disease burden.	[139]
Gaiser et al. (2015)	Evaluating blood levels of neuron specific enolase, chromogranin A, and circulating tumor cells as Merkel cell carcinoma biomarkers	CTCs	Stage I–IV	30	Maintrac	Correlation between CTC detection and disease outcomes.	[140]
Samimi et al. (2016)	Prognostic value of antibodies to Merkel cell polyomavirus T antigens and VP1 protein in patients with Merkel cell carcinoma	Anti-MCPyV-antibody	Stage I–IV	143	ELISA	Basal level of anti-VP1 antibodies used as prognostic marker. Anti-T-antigen antibodies are marker of disease recurrence or progression if detected >12 months after diagnosis	[141]
Fan et al. (2018)	Circulating cell-free miR-375 as surrogate marker of tumor burden in Merkel cell carcinoma	miRNA	Stage I–IV	102	RT-qPCR	Circulating miR-375 is a useful biomarker for tumor burden, therapy monitoring and follow-up of patients with MCC.	[142]
Riethdorf et al. (2019)	Detection and characterization of circulating tumor cells in patients with Merkel cell carcinoma	CTCs	Stage I–IV	51	CellSearch—CXC kit	Correlations between CTC counts and MCC aggressiveness.	[143]
Boyer et al. (2020)	Circulating tumor cell detection and polyomavirus status in Merkel cell carcinoma	CTCs	Stage I –IV	19	•CellSearch—CTC kit •RosetteSep/DEPArray	CTC presence associated with tumor stage and number of organs with metastases.	[144]

CTC: circulating tumor cell, OS: Overall Survival, MCPyV: Merkel Cell Polyomavirus, ctDNA: circulating tumor DNA, miRNA: microRNA, ELISA: Enzyme-linked immunosorbent assay, RT-qPCR: Reverse-transcriptase Polymerase Chain Reaction.
